# The antidiabetic compound 2-dodecyl-6-methoxycyclohexa-2,5-diene-1,4-dione, isolated from *averrhoa carambola* L., demonstrates significant antitumor potential against human breast cancer cells

**DOI:** 10.18632/oncotarget.4475

**Published:** 2015-06-15

**Authors:** Ying Gao, Renbin Huang, Yixuan Gong, Hyo Sim Park, Qingwei Wen, Nadin Marwan Almosnid, Uma D. Chippada-Venkata, Najlaa Abdulrhman Hosain, Eric Vick, Anthony Farone, Elliot Altman

**Affiliations:** ^1^ Tennessee Center for Botanical Medicine Research and the Department of Biology, Middle Tennessee State University, Murfreesboro, Tennessee, USA; ^2^ Pharmaceutical College, Guangxi Medical University, Nanning, Guangxi, PR China; ^3^ Division of Hematology and Medical Oncology, The Tisch Cancer Institute, Mount Sinai School of Medicine, New York, New York, USA

**Keywords:** antitumor, apoptosis, cell cycle, reactive oxygen species, NF-κB

## Abstract

2-Dodecyl-6-methoxycyclohexa-2,5-diene-1,4-dione (DMDD) is a cyclohexanedione found in the roots of *Averrhoa carambola* L., commonly known as starfruit. Researchers have shown that DMDD has significant therapeutic potential for the treatment of diabetes; however, the effects of DMDD on human cancers have never been reported. We investigated the cytotoxic effects of DMDD against human breast, lung and bone cancer cells *in vitro* and further examined the molecular mechanisms of DMDD-induced apoptosis in human breast cancer cells. DMDD suppressed the growth of breast carcinoma cells, but not normal mammary epithelial cells, via induction of G1 phase cell cycle arrest, oxidative stress and apoptosis. DMDD increased the level of intracellular reactive oxygen species (ROS) and DMDD-induced ROS generation was found to be associated with the mitochondrial activity. The cytotoxicity that was induced by DMDD was attenuated by co-treatment with the antioxidant N-acetyl-L-cysteine (NAC). DMDD-induced cell apoptosis involved the activation of both the intrinsic mitochondrial pathway and the extrinsic receptor pathway. In addition, DMDD inhibited the canonical NF-κB signaling pathway at all steps, including TNF-α production, phosphorylation of NF-κB p65 and IκBα, as well as TNF-α activated NF-κB p65 nuclear translocation. Collectively, our studies indicate that DMDD has significant potential as a safe and efficient therapeutic agent for the treatment of breast cancer.

## INTRODUCTION

Human cancers are one of the leading causes of death worldwide and breast cancers cause the highest mortality in women amongst cancers. It is estimated that 232,670 new cases of breast cancer were diagnosed in the US in 2014 and breast cancer represents 14.0% of all new cancer cases in the U.S. [1]. Current therapeutic treatments for cancer usually cause serious side effects, such as bladder, kidney, lung or heart damage. Thus the development of effective drugs with less adverse effects for the chemopreventive intervention of cancers is the top priority in cancer research.

**Figure 1:**
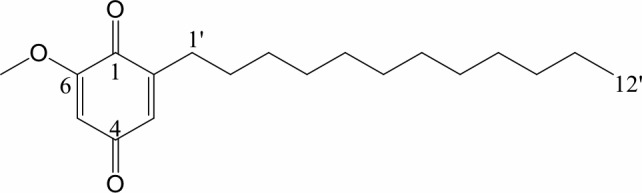
Chemical structure of DMDD

*Averrhoa carambola L.* (Oxalidaceae) is a perennial herb widely distributed in Southeast Asia. Its roots have been employed in Traditional Chinese Medicine (TCM) for thousands of years as a remedy for arthralgia and chronic paroxysmal headaches. Previously, a cyclohexanedione, 2-Dodecyl-6-methoxycyclohexa-2,5-diene-1,4-dione (DMDD) (Figure 1), was isolated from the roots of *Averrhoa carambola L.* and found to exhibit hypoglycemic and anti-lipid peroxidative effects in diabetic mice [2, 3, 4]. Apart from their use as pesticides as well as synthetic precursors to many organic compounds, cyclohexanediones and their derivatives have also attracted considerable attention because of their broad range of biological properties such as antimicrobial, antimalarial, and antitumor activities [5, 6, 7, 8]. However, the effects of DMDD on human cancers have not yet been investigated.

Because of recent studies linking diabetes and breast cancer [9, 10, 11, 12] and the fact that the antidiabetic drug metformin can effectively treat breast cancer [13, 14], we decided to investigate whether DMDD could be used as an antitumor agent against breast cancer. Tumor cells have a myriad of aberrant physiological properties compared to normal healthy cells and these differences have been targeted in the development of anticancer agents. Potential anticancer therapeutics have included agents that can induce apoptosis, increase oxidative stress, inhibit the global transcriptional regulator nuclear factor-kappa B (NF-κB), or suppress the relaxed cell cycle of cancer cells.

Numerous studies have shown that a wide range of anticancer agents induce apoptosis in cancer cells *in vitro*. Apoptosis, or programmed cell death, is crucial for the elimination of abnormal cells and the prevention of tumorigenesis [15]. Apoptosis involves two major pathways, the extrinsic and intrinsic pathways [16]. The extrinsic pathway, also known as the death receptor pathway, is triggered by a death ligand binding to a death receptor, such as tumor necrosis factor alpha (TNF-α). The extrinsic pathway initiates the caspase-8 dependent signal cascade. The intrinsic pathway, also known as the mitochondrial pathway, involves the release of apoptotic proteins, such as cytochrome *c* from mitochondria. Cytochrome *c* recruits Apaf-1 and caspase-9 and forms an apoptosome complex, which subsequently cleaves caspase-9. Crosstalk can occur between the intrinsic and extrinsic pathways. Both pathways activate caspase-3, -6 and -7, and induce a variety of cellular events including proteolysis and DNA fragmentation, which causes cell death [15, 16].

Oxidative stress is an imbalance between the production of free radicals, referred to as oxidants or reactive oxygen species (ROS), and a cell's ability to eliminate them by protective mechanisms is crucial for its survival [17]. Oxidative stress often occurs from exposure to ultra-violet (UV) light, environmental stress, or toxins. When cells undergo oxidative stress, ROS accumulate in the cells and damage intracellular molecules including proteins, lipids, DNA and RNA [18]. Studies have shown that oxidative stress plays a crucial role in a number of conditions such as vascular disease, neurodegeneration, anemia, auto-immune diseases, inflammatory responses and cancer [19, 20]. ROS levels have opposing effects: ROS activation below a specific threshold promotes cell survival; however, excessive ROS are known to be toxic, leading to cell death [21]. It is well established that oxidative stress induced by cancer therapy is essential to fight cancers. Examples of chemotherapeutic treatments that increase ROS are paclitaxel, doxorubicin, and cisplatin [22].

Nuclear factor-kappa B (NF-κB) is a transcription factor that plays a critical role across many cellular processes including embryonic and neuronal development, immune responses to infection, inflammation, cell proliferation, apoptosis and tumorigenesis [23, 24, 25]. Because the NF-κB pathway regulates the transcription of anti-apoptotic and cell proliferation genes, it is often critical for the survival of cancer cells. There has been increasing interest in targeting the NF-κB signaling pathway as a therapeutic option for cancer treatments. A variety of widely used anticancer agents suppress proliferation and induce apoptosis of various cancer cells by regulating NF-κB activities [23, 26].

In the present study, we demonstrated that the cyclohexanedione DMDD dramatically inhibits the proliferation of human breast, lung and bone cancer cells *in vitro*. We further investigated the molecular mechanism of DMDD's anti-proliferation effects, and demonstrated that DMDD suppresses the growth of breast carcinoma cells via induction of G1 phase cell cycle arrest, apoptosis, oxidative stress, and inhibition of the NF-κB signaling pathway.

## RESULTS

### DMDD exhibits anti-proliferation activity in human breast, lung and bone cancer cells

Because the antidiabetic drug metformin is known to be effective against breast cancer, we first evaluated the anti-proliferation activity of DMDD in different types of human breast carcinoma cell lines (MCF-7, BT20, MDA-MB-231) and in the normal human mammary epithelial cell lines (HMEC and MCF10A) as controls. To test whether DMDD might be effective against other cancers as well, we also tested DMDD in the human lung carcinoma cell line A549 and the human osteosarcoma cell line U2OS and in the normal human peripheral lung epithelial cell line HPL1A and the normal human primary umbilical vein endothelial cell line HUVEC as controls. We observed a concentration dependent anti-proliferation effect of DMDD in all tested cancerous cells after 48 h treatment, with IC_50_ values ranging from 3.13 μM to 5.57 μM (Table 1). In contrast, DMDD exhibited significantly less cytotoxicity against normal cells, especially mammary epithelial cells, with IC_50_ values ranging from 9.0 to 48.8 μM. It is notable that treatment with 3.2 μM DMDD caused a 25-57% growth inhibition of all of the cancerous cells that were tested, but had no effect on any of the normal cells that were tested.

**Table 1:** IC_50_ (μM) values of DMDD in human cancer cell lines and the normal cell line *in vitro*

Cell line	Description	IC_50_(μM)
MCF-7	Human breast carcinoma	3.44
BT20	Human breast carcinoma	5.03
MDA-MB-231	Human breast carcinoma	4.16
A549	Human lung carcinoma	5.57
U2OS	Human osteosarcoma	3.13
HMEC	Human mammary epithelial	48.8
MCF-10A	Human mammary epithelial	14.3
HPL1A	Human peripheral lung epithelial	11.5
HUVEC	Human primary umbilical vein endothelial	9.0

Different concentrations of DMDD were used to treat the cells for 48 h and cell viability was assessed using a PrestoBlue fluorescence assay. Values are means ± SD of three independent experiments.

A morphological examination of the cancerous cells was performed using an inverted microscope. MCF-7, BT20, MDA-MB-231, A549 and U2OS cells treated with DMDD at various concentrations (3.2-100 μM) for 24 and 48 h showed significant morphological changes indicative of apoptosis, such as cell shrinkage and reduced cell density (data not shown). Because DMDD was most effective and selective against breast cancer cells, we chose to proceed with determining the mechanism of action of DMDD in breast cancer cells.

### DMDD causes nuclear condensation, increase of cell permeability and disruption of mitochondrial potential in breast cancer cells

**Figure 2:**
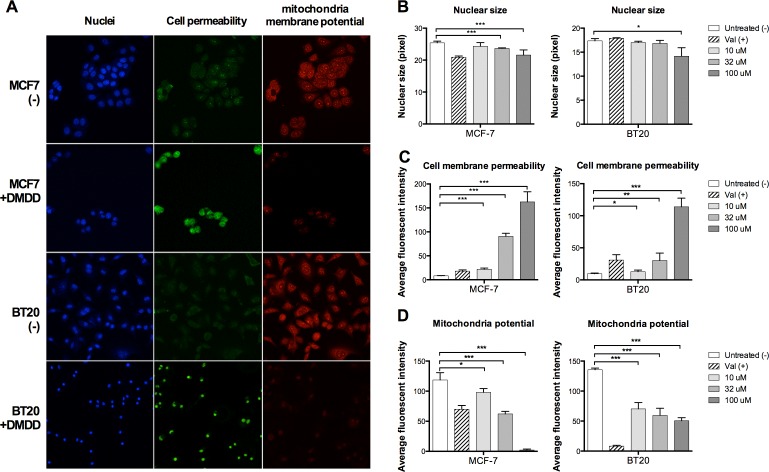
Multiplex HCS analysis of DMDD-induced cytotoxicity in MCF-7 and BT20 cells Cells were treated with different concentrations of DMDD for 24 h and the alteration in nuclear size, cell permeability, and mitochondrial membrane potential was simultaneously quantified by a HCS reader. **A.,** Representative cell images in three fluorescent-channels taken by the ArrayScan HCS reader. **B.**, **C.**, **D.**, Changes in nuclear size, cell permeability, and mitochondrial membrane potential in DMDD-treated MCF-7 and BT20 cells. Values are means ± SD of three independent experiments. *P ≤ 0.05; **P ≤ 0.01; ***P ≤ 0.001.

We examined the cellular changes of DMDD treated cells including nuclear morphology, cell membrane permeability and mitochondria membrane potential (ΔΨm), in parallel using high-content screening (HCS) analysis. The change of nuclear morphology was monitored by Hoechst dye staining. The cell membrane permeability was monitored by a permeability dye which only stains nuclei in permeabilized cells. Mitochondrial integrity was examined by a mitochondrial membrane potential dye which accumulates in healthy mitochondria with intact membrane potential, but not in depolarized mitochondria. Untreated MCF-7 and BT20 cells displayed normal sized nuclei, intact plasma membrane and brightly labeled mitochondria. After 24 h treatment with DMDD, the cells exhibited increased plasma membrane permeability as evidenced by higher green florescence intensity and loss of mitochondrial potential as evidenced by lower red florescence intensity, suggesting that they were undergoing severe cellular injury (Figure 2A). We observed dose-dependent effects of DMDD on membrane permeability and mitochondrial potential in MCF-7 and BT20 cells as shown in Figure 2C and 2D. In addition, MCF-7 treated with 33 and 100 μM DMDD and BT20 cells treated with 100 μM DMDD showed a significant decrease in nuclear size (***P < 0.001 or *P < 0.01) (Figure 2B).

### DMDD induces both the intrinsic and extrinsic apoptosis pathways in human breast cancer cells

**Figure 3:**
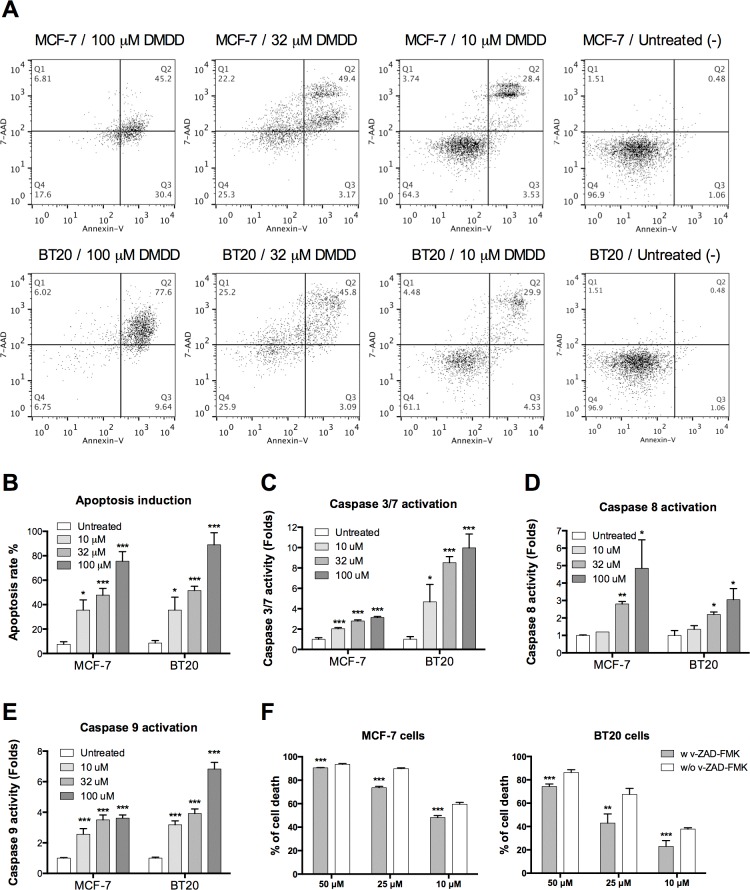
Evaluation of caspase-dependent apoptosis in human breast carcinoma cells MCF-7 and BT20 **A.**, Flow cytometry analysis of apoptosis induced by DMDD in MCF-7 and BT20 cells. **B.**, Percentage of apoptosis induced by different concentrations of DMDD in MCF-7 and BT20 cells. Cells were treated with different concentrations of DMDD for 48 h and assessed using Annexin V/7-AAD double staining. **C.**, **D.**, **E.**, Caspase 3/7, caspase 8, and caspase 9 activation in MCF-7 and BT20 cells treated with DMDD. Cells were treated with different concentrations of DMDD for 4 h and assessed using the Caspase-Glow 3/7, Caspase-Glow 8, or Caspase-Glow 9 assay. Cells treated with DMSO only served as the negative control, and fold-induction relative to the negative control is shown. **F.**, DMDD induces caspase-dependent cell death in MCF-7 and BT20 cells. MCF-7 and BT20 cells were treated with the general caspase inhibitor z-VAD-FMK (100 μM) for 1 h, and treated with 10, 25, 50 μM of DMDD or the DMSO-only control for 24 h before the viability test. The cell viability was determined using the PrestoBlue assay. The error bars indicate the standard deviation from three independent experiments. *P ≤ 0.05; **P ≤ 0.01; ***P ≤ 0.001.

We employed the Annexin V/7-AAD double staining assay to confirm that DMDD promoted apoptosis in MCF-7 and BT20 cells. We found that DMDD induced apoptosis in MCF-7 and BT20 cells (Figure 3A) and that the apoptosis induction was concentration-dependent (Figure 3B).

Next, we evaluated caspase 8, an early apoptotic marker and initiator of the extrinsic apoptosis pathway; caspase 9, an initiator of the intrinsic apoptosis pathway; as well as caspase 3/7, the executioner caspase that triggers apoptosis [28], using luminescence assays. As shown in Figure 3C, 3D and 3E, in MCF-7 and BT20 cells treated with DMDD for 4 h, the activities of caspase-3/7, -8, and -9 increased significantly in a concentration-dependent manner compared to untreated cells (*P < 0.05, **P < 0.01 or ***P < 0.001). To further confirm the caspase-dependency of DMDD-induced cell death, we pre-treated MCF-7 and BT20 cells with the general caspase inhibitor, carbobenzoxy-valyl-alanyl-aspartyl-[O-methyl]-fluoromethylketone (z-VAD-FMK), and found that 100 μM z-VAD-FMK exhibited significant protection against cell death induced by 10-50 μM DMDD (**P < 0.01 or ***P < 0.001) (Figure 3F). Pretreatment with 100 μM of z-VAD-FMK could not attenuate the cytotoxicity induced by 100 μM of DMDD (data not shown).

**Figure 4:**
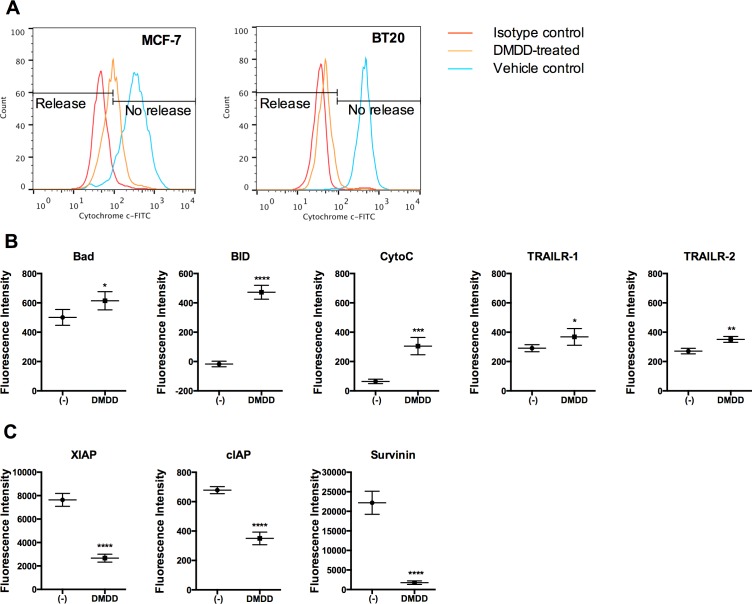
Analysis of cytochrome c release and apoptotic protein expression in DMDD-treated BT20 cells **A.**, Induced cytochrome *c* release by DMDD. The red curve shows the isotype control, the blue curve shows the negative control (DMSO only), and the yellow curve shows the treated samples. Cells were treated with different concentrations of DMDD for 24 h, and the release of cytochrome *c* was assessed by flow cytometry using anti-Cytochrome *c*-FITC staining. **B.**, Up-regulated apoptotic proteins by DMDD. **C.**, Down-regulated apoptotic proteins by DMDD. BT20 cells were treated with 50 μM DMDD for 6 h. 50 μg of cell lysate from both treated and non-treated BT20 cells were incubated 2 h at RT with a Human Apoptosis Array Chip. The antibody chip was then washed and biotin-conjugated antibody cocktail was added to detect the apoptosis-related proteins. After incubation with fluorescence-conjugated streptavidin, the fluorescent signals were detected. The error bars indicate the standard deviation from two independent experiments carried out in replicate (*n* = 4). *P ≤ 0.05; **P ≤ 0.01; ***P ≤ 0.001, ****P ≤ 0.0001.

To clarify whether DMDD also induced the intrinsic apoptotic pathway, we determined whether cytochrome *c* was released from the mitochondria of the treated cells. As shown in Figure 4A, cytochrome *c* was released from the MCF-7 and BT20 cells treated by DMDD for 24 h. We also investigated the regulation of apoptotic proteins using a human apoptotic protein array. The protein expression profile revealed that the death receptors TNF-α-related apoptosis-inducing ligand receptor 1 and 2 (TRAIL-R1 and TRAIL-R2), the pro-apoptotic proteins Bcl-2-associated death promoter (Bad) and BH3 interacting-domain death agonist (BID), are significantly up-regulated by DMDD (*P < 0.05, **P < 0.01, or ****P < 0.0001) (Figure 4B). Meanwhile, the apoptosis inhibitors, cellular inhibitor of apoptosis (cIAP), X-linked inhibitor of apoptosis protein (XIAP) and Survivin are significantly down-regulated by DMDD (****P < 0.0001) (Figure 4C).

### DMDD induces oxidative stress in human breast cancer cells

**Figure 5:**
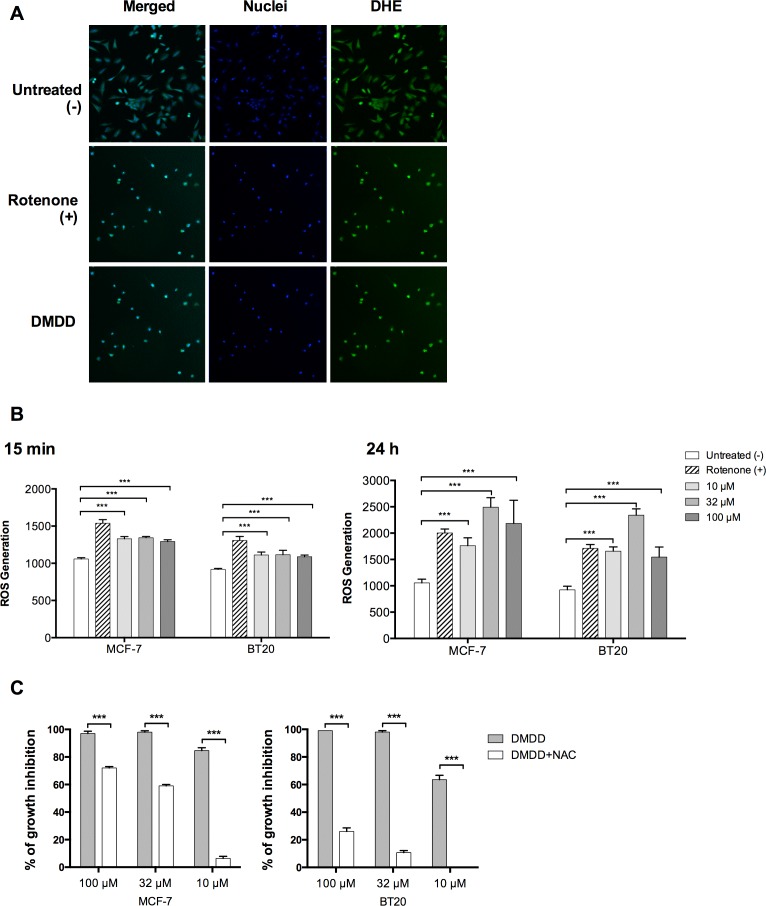
Oxidative stress induction in human breast carcinoma cells MCF-7 and BT20 Cells were treated with different concentrations of DMDD for 24 h, and the ROS level was assessed using DHE staining on a HCS reader. **A.**, Typical HCS images obtained for oxidative stress assay in BT20 cells. **B.**, Quantitative ROS levels from HCS images. The error bars indicate the standard deviation from three experiments. *P ≤ 0.05; **P ≤ 0.01; ***P ≤ 0.001. **C.,** Co treatment of MC-7 or BT20 cells with the antioxidant N-acety1-L-cysteine (NAC) and different concentrations of DMDD.

We determined the effect of DMDD on cellular ROS levels in MCF-7 and BT20 cells using HCS and an oxidative stress assay. The generation of ROS in cells was quantified by the oxidation of non-fluorescent dihydroethidium (DHE) to fluorescent ethidium. As shown in Figure 5B, a marked and immediate increase in ROS generation was detected after 15 min of DMDD treatment in both MCF-7 and BT-20 cells and after 24 h of DMDD treatment even higher ROS levels were detected in both MCF-7 and BT-20 cells, An intermediate concentration of DMDD (32 μM) enhanced ROS levels nearly three-fold versus the non-treated control, while high and low concentrations (100 μM and 10 μM) of DMDD enhanced ROS nearly two-fold versus the untreated control (Figure 5A and 5C). To further investigate whether DMDD-induced cytotoxicity was associated with ROS levels, we co-treated MCF-7 or BT20 cells with the antioxidant N-acetyl-L-cysteine (NAC) and different concentrations of DMDD for 48 h. We observed that 10 mM NAC attenuated DMDD-induced cell death of breast cancer cells in a concentration-dependent manner (Figure 5C). It is notable that NAC almost completely reversed the cytotoxic effect caused by 10 μM DMDD in both MCF-7 and BT20 cells.

### DMDD arrests the cell cycle of cancer cells at G1 phase

**Figure 6:**
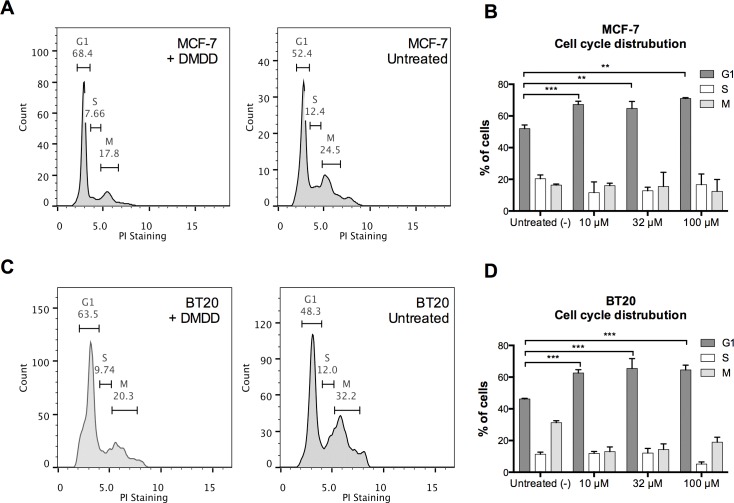
Cell cycle distribution of human breast carcinoma cells MCF-7 and BT20 treated with DMDD Cells were synchronized for 24 h prior to the treatment with different concentrations of DMDD for 24 h and assessed using propidium iodide (PI) staining. **A.**, **B.**, The cell cycle distribution of MCF-7 cells treated with DMDD. **C.**, **D.**, The cell cycle distribution of BT20 cells treated with DMDD. The error bars indicate the standard deviation from three experiments. *P ≤ 0.05; **P ≤ 0.01; ***P ≤ 0.001.

To examine if DMDD altered the distribution of the cell cycle, we performed a cell cycle analysis with MCF-7 and BT-20 cells. As shown in Figure 6, DMDD induced a significant increase in the cell number at the G1 phase after treatment for 24 h, with a corresponding decrease in the S and G2-M phases in both breast cancer cell lines. In MCF-7 cells, the percentage of G1 phase cells increased from 52±2% to 67±2% at 10 μM concentration; and in BT20 cells, the percentage of G1 phase cells increased from 46±1% to 63±2% at 10 μM concentration. These results suggest that DMDD disrupts the G1-S transition during cell division. The alteration of the cell cycle did not show obvious concentration-dependency.

### DMDD inhibits TNF-α production in LPS induced THP-1 cells

**Figure 7:**
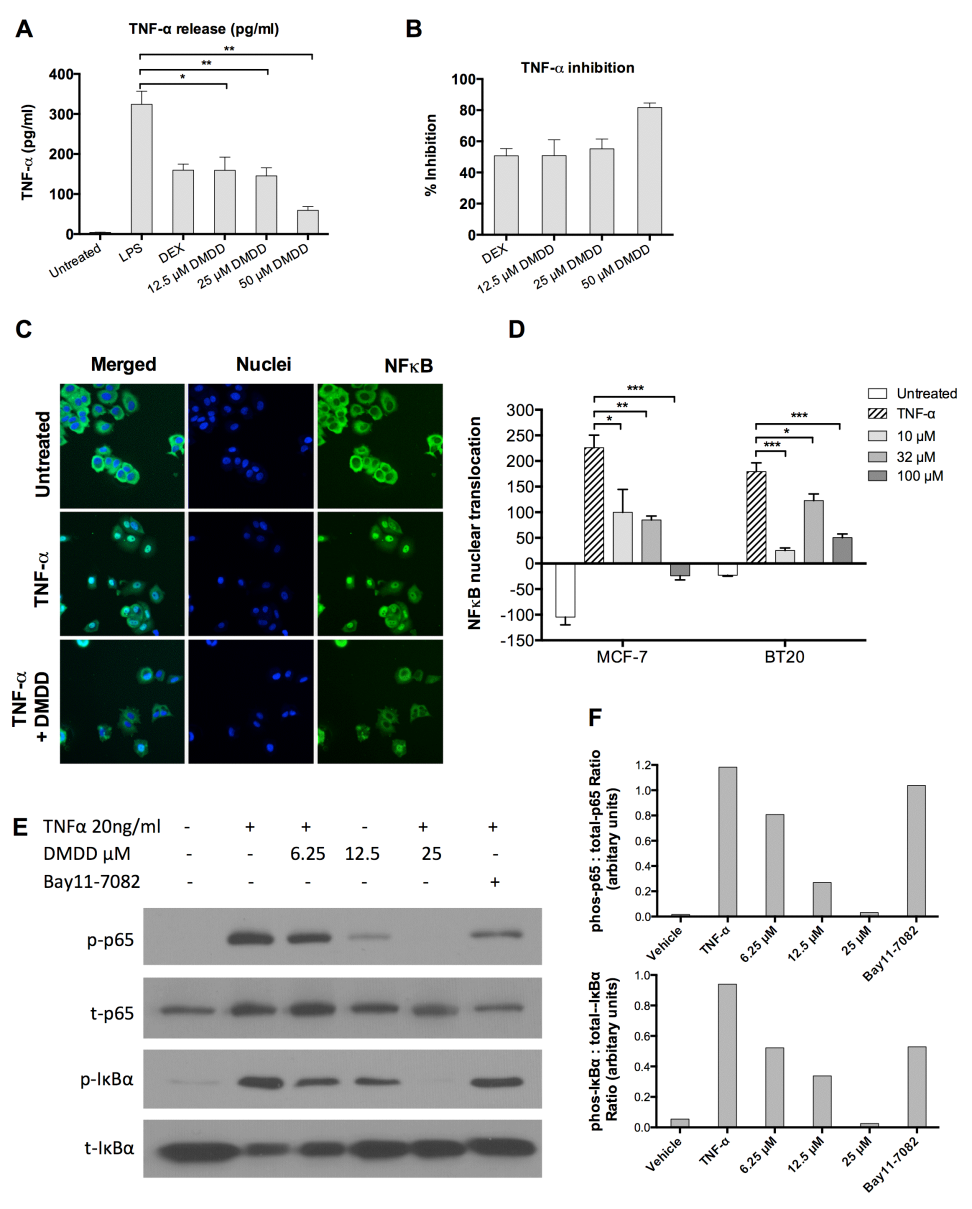
DMDD inhibits LPS-induced TNF-α production and suppresses the canonical NF-κB signaling pathway **A.**, TNF-α production at pg/ml in DMDD-treated THP-1 cells. **B.**, Relative percent inhibition of TNF-α by DMDD. PMA (100 nM)-treated THP-1 cells were pretreated with 1 μM dexamethasone (DEX) or various concentrations of DMDD for 30 min followed by stimulation with 100 ng/ml LPS for 4 h. 1 uM of DEX was used as a positive control for TNF-α inhibition. Supernatants were collected and the TNF-α levels were measured using a human TNF-α ELISA kit. **C.**, Representative HCS images of the NF-κB translocation in MCF-7 cells. **D.**, Values of NF-κB nuclear translocation in MCF-7 and BT20 cells. Cells were treated with different concentration of DMDD for 2 h, followed by stimulation with 10 ng/ml TNF-α for 30 min. Cells were sequentially stained with NF-κB p65 primary antibody, DyLight 488-conjugated secondary antibody and Hoechst 33342 dye, and measured with an ArrayScan HCS reader. Cells treated with TNF-α alone served as the negative control. **E.**, **F.**, Immunoblotting of phospho-NF-κB p65, total NF-κB p65, phospho-IκBα, and total IκBα in MCF-7 cells. Cells were treated with different concentrations of DMDD for 3 h, followed by stimulation with 20 ng/ml TNF-α for 15 min, and protein expression was detected by western blotting analysis. The error bars indicate the standard deviation from three independent experiments. *P ≤ 0.05; **P ≤ 0.01; ***P ≤ 0.001.

To determine whether DMDD affected TNF-α production in LPS-induced THP-1 human monocytic leukemic cells, a human TNF-α ELISA was conducted to assess the level of TNF-α in the supernatant. 12.5-50 μM DMDD did not affect the viability of THP-1 cells after treatment with or without LPS stimulation (data not shown). As shown in Figure 7A and 7B, DMDD caused a significant reduction of the TNF-α level in the supernatants of the cell cultures relative to the cells treated with LPS alone (*P < 0.05 or **P < 0.01). 12.5, 25 and 50 μM DMDD reduced TNF-α production by 50.8%, 55.2% and 81.7% compared with the negative control, respectively

### DMDD suppresses the canonical NF-κB signaling pathway

We assessed the ability of DMDD to inhibit NF-κB activation induced by the inflammatory cytokine TNF-α by using anti-NF-κB p65 antibody conjugated with a fluorescent dye to monitor the NF-κB translocation from the cytoplasm to nuclear region. As shown in Figure 7C, in non-treated MCF-7 cells, a high fluorescence intensity of NF-κB was found in the cytoplasm, but rarely in the nuclei, indicating that NF-κB is not activated under normal conditions. Following stimulation with TNF*-*α, the NF-κB fluorescent intensity significantly increased in the nuclear region, indicating NF-κB translocation from the cytoplasm to the nucleus. We observed significant inhibition of TNF*-*α-induced NF-κB nuclear translocation in both MCF-7 and BT20 cells treated with DMDD as evidenced by decreased intranuclear NF-κB fluorescence intensity (*P < 0.05, **P < 0.01 or ***P < 0.001) (Figure 7D). In MCF-7 cells, concentration-dependent inhibition was observed; however, in BT20 cells, the lowest concentration (10 μM) yielded more significant inhibition than medium (32 μM) and high (100 μM) concentrations.To further investigate the mechanism of DMDD suppression of NF-κB activation, we examined the effects of DMDD on TNF-α stimulated phosphorylation of NF-κB p65 and IκBα. As shown in Figure 7E, TNF-α stimulated strong phosphorylation of NF-κB p65 and IκBα, and the effect was suppressed by the treatment with DMDD in a dose-dependent manner. These results suggest that DMDD suppresses the NF-κB canonical signaling pathway.

## DISCUSSION

In this study, we demonstrated that the cyclohexanedione DMDD potently inhibited the growth of the human carcinoma cell lines (MCF-7, BT20, MDA-MB-231, A549 and U2OS), while causing notably less toxicity in the normal epithelial and endothelial cell lines (HMEC, MCF-10A, HPL1A and HUVEC). Collectively, our results indicate that DMDD is selective against cancer cells, especially breast cancer cells. Consistent with our *in vitro* findings, Zheng et al. [4] also showed that administration of a single dose of DMDD at 5,000 mg/kg body weight did not cause any acute toxicity *in vivo*.

The cytotoxic effect of DMDD on breast cancer cells was accompanied by the induction of apoptosis. In addition to nuclear condensation and mitochondrial permeability changes shown by high-content screening (HCS) multiplex cytotoxicity analysis, the dose-dependent apoptosis induction was further confirmed by Annexin V/7-AAD double staining and a marked and rapid activation of several apoptosis-inducing caspases (−3/7, −8 and −9).

Apoptosis can be induced by either the intrinsic or the extrinsic pathway. We found that both pathways were activated during DMDD-induced cell death. Caspase-8, the initiator caspase of the extrinsic pathway, was activated by DMDD and the death receptors TRAIL-R1 and TRAIL-R2 were upregulated in an apoptotic antibody array. We also showed that DMDD-induced apoptosis was accompanied by cytochrome *c* release, the hallmark of apoptosis induction by the intrinsic pathway [29, 30]. Several studies have demonstrated evidence of cross talk between extrinsic and intrinsic pathways in apoptosis [31, 32]. In the extrinsic pathway, once caspase-8 is activated, it can either directly cleave caspase-3 or cleave BID, which subsequently translocates to the mitochondrial membrane, disrupts the mitochondrial transmembrane potential and releases cytochrome *c* [33]. Therefore, it's not surprising that DMDD activated both signaling pathways in breast cancer cells. More importantly, we showed that pretreatment with the general caspase inhibitor z-VAD-FMK rescued the cell viability of DMDD-treated MCF7 and BT20 cells, thus confirming the caspase-dependence of DMDD-induced cell death.

Mitochondria are one of the main sources of ROS in mammalian cells and the generation of ROS has been suggested to be one of the causes of mitochondrial disruption [34, 35, 36]. Evidence has also shown that ROS are common mediators of apoptotic cell death [21]. In the apoptotic process, initial stress-induced damage does not kill cells directly; rather it triggers an apoptotic signaling program that leads to cell death [37]. DMDD treatment immediately induced a sustained ROS overproduction in MCF-7 and BT20 cancer cells and co-treatment with the general antioxidant NAC almost completely blocked DMDD-induced cell death, suggesting that DMDD-induced oxidative stress is the underlying mechanism of cytotoxicity. The elevation of intracellular ROS appears to be an upstream signal that initiates the series of apoptotic events induced by DMDD. Moreover, oxidative stress is known to inhibit the transition of cells from G1 to the S phase by oxidation of membrane lipids that prolong the G1 phase [21]. This is consistent with our finding that DMDD inhibits the cell cycle transition from G1 to the S phase in the cancer cells.

NF-κB has been associated with cancer due to its ability to create a positive feedback loop of inflammation [25]. While NF-κB is dormant in the cytosol of normal cells, many cancer cells have been shown to possess constitutively active NF-κB, either due to mutations in the NF-κB inhibitor IκB, or in NF-κB itself that allow for its continuous activation [38]. Without regulation, this ongoing activation enhances cancer cell proliferation. Inhibition of NF-κB activation blocks cancer cell proliferation and therefore targeting the NF-κB signaling pathway has become an important therapeutic option for cancer treatments. NF-κB may be activated from its inactive precursors by two signaling pathways: the canonical pathway (or classical pathway), which is activated in the presence of inflammatory cytokines or compounds, and the noncanonical pathway (or alternative pathway) which is activated during B cell development [23]. The canonical pathway is most usually associated with cancer, and is mainly activated by TNF-α, IL-1, and LPS [39]. In the canonical pathway, IκBα is phosphorylated by IKK, resulting in the nuclear translocation of p65-containing heterodimers [40]. Our results showed that DMDD not only significantly reduced the TNF-α level in cells treated with LPS, but also dramatically inhibited the phosphorylation of IκBα and NF-κB p65 as well as NF-κB p65 translocation from the cytoplasm to the nuclei. These results suggest that DMDD serves as an inhibitor of the canonical NF-κB pathway in human breast carcinoma cells and can potentially prevent the initial inflammatory cascade in cancer cells that drives further proliferation.

How DMDD-induced oxidative stress is involved in the negative regulation of NF-κB pathway is an interesting question. ROS can regulate NF-κB activation both positively or negatively through various mechanisms that are dependent on the cell type [41, 42, 43]. It is known that sustained oxidative stress can inhibit NF-κB activation by glutathionylation of IκBα [43] or by inactivating the proteasome [44]. Thus DMDD-induced sustained oxidative stress can potentially prevent NF-κB pathway activation through the above mentioned mechanisms. In future experiments, the detailed mechanism of DMDD-induced NF-κB inactivation and its regulation by ROS will be investigated.

## CONCLUSION

**Figure 8:**
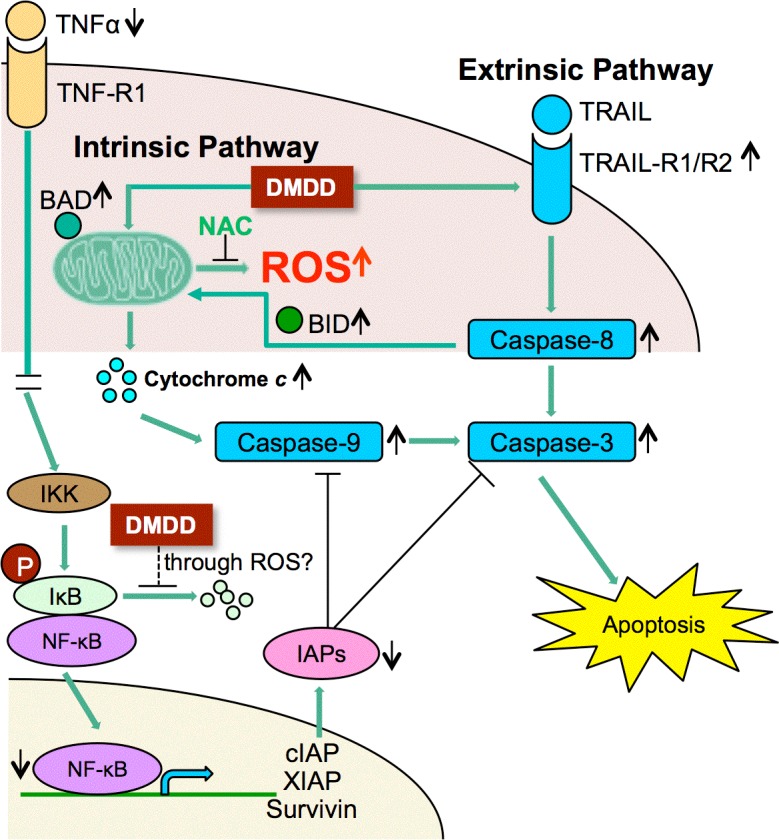
Potential scheme of intrinsic and extrinsic apoptotic pathway induction by DMDD ↑Up-regulation; ↓Down-regulation.

DMDD induced apoptosis in breast cancer cells through the generation of intracellular ROS and the inhibition of NF-kB activation. DMDD-induced apoptosis in breast cancer cells involved both the extrinsic and intrinsic signaling pathways and DMDD inhibited the activation of each step of the canonical NF-κB signaling pathway as shown in Figure 8. DMDD affects the extrinsic apoptotic pathway by suppressing TRAIL receptors and caspase 8, and affects the intrinsic apoptotic pathway by damaging mitochondrial membrane potential, releasing cytochrome *c* and activating caspase 9 and caspase 3/7. DMDD also inhibits the production of TNF-α and suppresses the phosphorylation of IκBα, which leads to IκBα degradation and release of NF-κB. DMDD further inhibits NF-κB phosphorylation and translocation to the nucleus, which subsequently inhibits transcription of target genes including anti-apoptotic proteins such as cIAP, XIAP and Survivin. The ability of DMDD to inhibit the growth of human breast carcinoma cells without severe toxicity to normal cells indicates that DMDD is a promising candidate for cancer treatment, and results from this study suggest a potential important mechanism of DMDD for cancer therapies.

## MATERIALS AND METHODS

### Plant materials and extraction and isolation of DMDD

The plant material *Averrhoa carambola* L. was collected from Linshan County, Guangxi Autonomous Region, China, in June 2010 and was identified by Prof. Lai Mao-xiang. The collections were carried out on private land under the permission of the land owner. The voucher specimen (No. 20100605) was deposited in the Guangxi Institute of Chinese Medicine & Pharmaceutical Science herbarium (Guangxi, China). The isolation of DMDD was performed as described by Zheng et al. [4]. DMDD stock solutions were prepared by dissolving DMDD in dimethyl sulfoxide (DMSO) at a concentration of 10 mM. In all tests, the DMDD stock solution was further diluted in cell culture medium by 100 fold or more, and 1% DMSO was used as a vehicle control in the untreated cells.

### Cell culture

BT20, A549, and THP-1 (ATCC, USA) were maintained in RPMI 1640 medium (Sigma-Aldrich, USA). MCF-7 and MDA-MB-231 (ATCC, USA) were maintained in Dulbecco's Modified Eagle Medium (DMEM) with 2 mM L-glutamine (Sigma-Aldrich, USA). HMEC and U2OS (ATCC, USA) were maintained in McCoy's 5A medium (ATCC, USA). MCF-10A (ATCC, USA) was maintained in MEBM basal medium (Lonza corporation, USA) supplemented with the MEGM SingleQuot Kit (Lonza corporation, USA) and 100 ng/ml cholera toxin (Sigma-Aldrich, USA). HPL1A was obtained from Nagoya University, Japan, and maintained in DMEM/F-12K medium (Sigma-Aldrich, USA). HUVEC (ATCC, USA) was maintained in Vascular Cell Basal Medium (ATCC, USA) supplemented with the Endothelial Cell Growth Kit-BBE (ATCC, USA). All mediums were supplemented with 10% fetal bovine serum (FBS) (Life Technologies, USA) and 1% penicillin-streptomycin (Sigma-Aldrich, USA) and all cells were incubated in a humidified atmosphere with 5% CO_2_ at 37°C.

### Anti-proliferation assay and IC_50_ determination

The proliferation inhibition potential of DMDD was determined in human breast cancer cell lines MCF-7 and BT20 by a fluorescence dye staining method with the normal cell line HMEC serving as a control. Cells were seeded at a density of 4∼5 ×10^3^ cells/well in a 96-well tissue culture-treated plate (Corning, USA) and were incubated overnight. They were then treated with DMDD at final concentrations of 320 μM, 100 μM, 32 μM, 10 μM, 3.2 μM, 1 μM, and 0.32 μM. An experimental control with cells only was also included. After incubation for 48 h, the viability of cells was assessed using PrestoBlue dye (Invitrogen) according to manufacturer's protocol. The fluorescence intensity was measured on a SpectraMax M5 microplate reader (Molecular Devices, USA) at a fluorescent excitation wavelength of 555 nm and emission wavelength of 590 nm. The results were expressed as a percentage, relative to untreated control cells, and the half maximal inhibitory concentration (IC_50_) values were calculated using non-linear regression analysis. For the caspase inhibitor assay, the cells were treated with 100 μM z-VAD-FMK (Sigma-Aldrich, USA) for 1 h, and treated with different concentrations of DMDD for 24 h before the viability test. For the NAC attenuation assay, the cells were treated with different concentrations of DMDD followed by incubation with or without 10 mM NAC (Sigma-Aldrich, USA) for 48 h, and then cell viability was assessed.

### Multi-parameter cytotoxicity measurement

MCF-7 and BT20 cells were seeded in a 96-well plate (Corning, USA) at a density of 4,000 cells/well and subsequently treated with different concentrations (10, 32, 100 μM) of DMDD. Cells treated with DMSO only and cells treated with 100 μM Valinomycin (Sigma-Aldrich, USA) served as negative and positive controls, respectively. After 24 h, the cells were stained with a mixture of fluorescent dyes including Hoechst 33342, cell permeability dye and mitochondrial membrane potential dye (Thermo Scientific, USA). These dyes indicated changes in nuclear morphology (Channel 1), cell membrane permeability (Channel 2) and mitochondrial trans-membrane potential (Channel 3), respectively. The cells were fixed and washed, and images for each fluoroprobe were acquired at different channels using suitable filters with a 20X objective and analyzed on the Arrayscan VTI HCS Reader (Thermo Scientific, USA). The Cell Health Profiling BioApplication (Thermo Scientific, USA) was used for image acquisition and analysis. For each well, at least 400 cells were automatically acquired and analyzed. The average fluorescent intensity was used to quantify changes in each channel.

### Apoptosis assay

MCF-7 and BT20 cells were seeded in a 96-well plate (Corning, USA) at a density of 8,000 cells/well and were subsequently treated with different concentrations (10, 32, 100 μM) of DMDD for 48 h. Afterwards, the cells were stained with Annexin V (Millipore, USA) and 7-Aminoactinomycin (7-AAD) (Millipore, USA), and analyzed on a Guava flow cytometer using InCyte software (Millipore, USA).

### Cytochrome c release assay

MCF-7 and BT20 cells were seeded in a 96-well plate (Corning, USA) at a density of 8,000 cells/well and were subsequently treated with 100 μM of DMDD for 24 h. The percentage of cells releasing cytochrome *c* from mitochondria was determined using the FlowCellect Cytochrome *c* Kit (Millipore, USA) according to the manufacturer's protocol. Briefly, cells were permeabilized, fixed, and stained with either anti-IgG1-FITC Isotype control or anti-Cytochrome *c*-FITC dye. Data was acquired and analyzed using a Guava flow cytometer and InCyte software (Millipore, USA).

### Caspase-3/7, -8, -9 activation assays

Caspase-3/7, -8, and -9 activities were analyzed using an *in-situ* luminescent maker in MCF-7 and BT20 cells respectively. Cells were seeded in a white-walled 96-well plate (Greiner, USA) and treated with different concentrations (10, 32, 100 μM) of DMDD for 4 h. Caspase activities were then determined using the Caspase-Glo 3/7 Assay (Promega, USA), Caspase-Glo 8 Assay (Promega, USA), or the Caspase-Glo 9 Assay (Promega, USA) according to the manufacturer's instructions. Briefly, equal volumes of Caspase-Glo 3/7, 8 or 9 reagents containing protease inhibitor MG-132 were added to the treated cells in a final volume of 200μl per well. Samples were incubated at room temperature for 1 h and the luminescence of each sample was measured using a SpectraMax M5 Microplate Reader (Molecular Devices, USA). Cells treated with DMSO only served as the negative control, and the data is shown as fold-induction relative to the negative control.

### Oxidative stress determination

MCF-7 and BT20 cells were seeded in a 96-well tissue culture-treated plate (Corning, USA) at a density of 8,000 cells/well and were subsequently treated with different concentrations (10, 32, 100 μM) of DMDD for 24 h. Cells treated with DMSO only served as the negative control, and cells treated with 1 μM Retenone (Sigma-Aldrich, USA) served as the positive control. Afterwards, the cells were stained with Hoechst 33342 (Thermo Scientific, USA) and dihydroethidium (DHE) (Sigma-Aldrich, USA), fixed and evaluated on an ArrayScan VTI HCS Reader (Thermo Scientific, USA). The generation of ROS in cells was quantified by the oxidation of non-fluorescent DHE to fluorescent ethidium, which subsequently binds to DNA. The Nuclear Translocation BioApplication software (Thermo Scientific, USA) was used for image acquisition and analysis. For each well, at least 400 cells were automatically acquired and analyzed. Oxidative stress activation was assessed by the DHE staining in the nucleus and measured by the average intensity of fluorescent ethidium in the identified nuclear region (MEAN_CircAvgIntenCh2).

### Cell cycle analysis

MCF-7 and BT20 cells were first synchronized in the G0 phase by culturing cells for 24 h in serum-free medium, and then treated with 100 μM DMDD for 24 h. Cell cycle analysis was performed using propidium iodide (PI) (Millipore, USA) staining as described [27]. Cells were analyzed using a Guava flow cytometer with InCyte software (Millipore, USA).

### Human TNF-α ELISA

THP-1 cells were seeded in a 12-well plate at a density of 5×10^5^ cells/well, and incubated with 100 nM phorbol-12-myristate-13-acetate (PMA) (Sigma-Aldrich, USA) for 72 h. After washing the wells with warm RPMI 1640 medium, cells were pretreated with various concentrations (12.5, 25, 50 μM) of DMDD for 30 minutes followed by stimulation with 100 ng/ml *Salmonella enterica* serotype typhimurium lipopolysaccharide (LPS) (Sigma-Aldrich, USA) for 4 h. Dexamethasone (Sigma-Aldrich, USA) at 1 μM was used as a positive control. Supernatants were collected for the quantification of human TNF-α using the Duoset enzyme-linked immunosorbent assay (ELISA) development kit (R&D Systems, USA).

### Inhibition assay of TNF-α activated NF-κB translocation

MCF-7 and BT20 cells were seeded in a 96-well plate (Corning, USA) at a density of 8,000 cells/well and then treated with various concentrations (10, 32, 100 μM) of DMDD for 2 h, followed by incubation with 10 ng/ml tumor necrosis factor-alpha (TNF-α) (Sigma-Aldrich, USA) for 30 min. Untreated cells and cells treated with 10 ng/ml TNF-α alone served as controls. Cells were fixed, permeabilized, and sequentially stained with NF-κB p65 primary antibody (Cell Signaling Technology, USA), DyLight 488-conjugated secondary antibody, and Hoechst 33342 dye. The Hoechst and DyLight fluorophores detect changes in nuclear morphology (blue fluorescence) and NF-κB distribution (green fluorescence), respectively. The samples were analyzed on an Arrayscan VTI HCS Reader (Thermo Scientific, USA). The Nuclear Translocation BioApplication (Thermo Scientific, USA) was used for image acquisition and analysis. For each well, at least 400 cells were automatically acquired and analyzed. The translocation index was calculated by measuring the average intensity difference of NF-κB between the identified cytoplasmic region and nuclear region (MEAN_CircRingAvgIntenDiffCh2).

### Western blotting analysis

MCF-7 cells were treated with various concentrations (6.25, 12.5, 25 μM) of DMDD for 3 h. Proteins were extracted from the cells using M-PER Mammalian Protein Extraction Reagent (Thermo Scientific, USA) supplemented with a protease inhibitor cocktail (Sigma, USA), separated on a Novex 4-20% Tris-glycine gel (Life technologies, USA), and electrophoretically transferred to nitrocellulose membranes. The membranes were blocked for 1 h in TBST buffer (50 mM Tris, 150 mM NaCl, 0.1% Tween 20, pH 7.9) with 5% fat-free milk at room temperature and then incubated overnight with primary antibodies at 4°C. After hybridization with horseradish peroxidase (HRP)-conjugated secondary antibody, protein bands were detected using the SuperSignal West Pico Chemiluminescent Substrate (Thermo Scientific, USA). IκBα, phosphorylated IκBα, NF-κB p65 and phosphorylated NF-kB p65 antibodies (Cell Signaling Technologies, USA) were the primary antibodies used in the NF-κB assay.

### Apoptosis antibody array

The Human Apoptosis Antibody Array Kit (RayBiotech, USA) was used to examine multiple apoptotic regulators according to the manufacturer's instructions. Briefly, 1 × 10^5^ cells were plated in each well of a 12-well plate and treated with 50 μM DMDD for 6 h. The cells were then lysed with Cell Lysis Buffer containing proteinase inhibitor from the kit. The cell lysate was concentrated using a protein concentration spin column (EMD Millipore, USA) to a 2 mg/ml concentration. After blocking, the cell lysate was incubated with the glass chip provided in the kit for 2 h at room temperature. Following five washes, the cocktail of biotin-conjugated antibodies was added to the glass chip and incubated overnight at 4°C. Afterwards, HiLyte Plus-conjugated streptavidin was added to the chip and incubated for 2 h at room temperature. Image scanning and data extraction was completed by RayBiotech. Fluorescent intensities from each dot of the array were normalized to the internal control.

### Statistical analysis

Statistical significance was calculated using the two-tailed Student's t test. *P ≤ 0.05; **P ≤ 0.01; ***P ≤ 0.001.
